# Supplementary material for: Altered Monocyte and Lymphocyte Phenotypes Associated with Pathogenesis and Clinical Efficacy of Progestogen Therapy for Peritoneal Endometriosis in Adolescents
Source: Cells. 2024 Jul 12;13(14):1187. doi: 10.3390/cells13141187 (PMC11274988; doi:10.3390/cells13141187)
Supplement: Supplementary file 1 [file cells-13-01187-s001.zip › cells-3001531-supplementary.pdf]

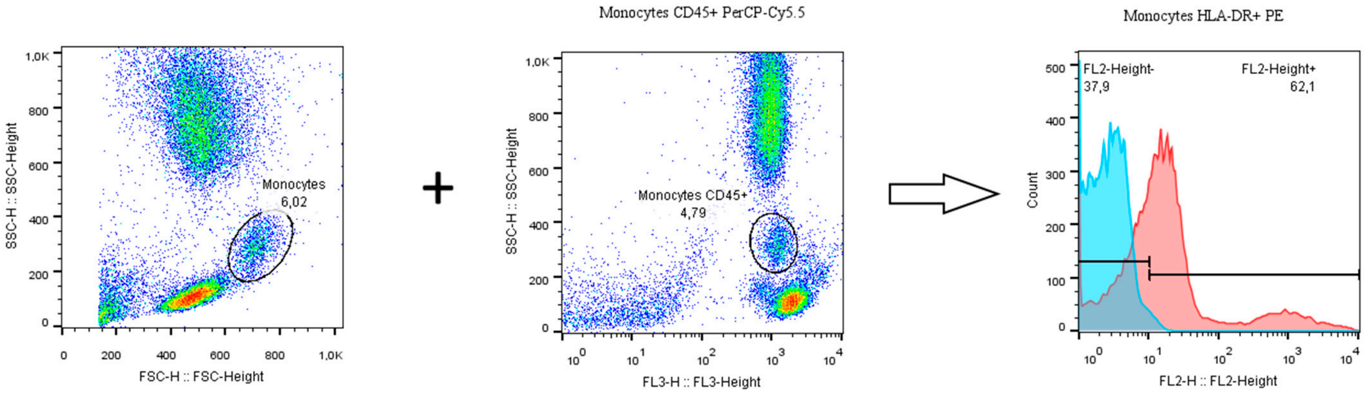

Figure S1. Gating strategy for monocytes on flow cytometry analysis. From SSC-FSC diagram monocytes subpopulation were chosen among with on SSC-CD45 diagram and then cells positive for markers – HLA-DR, CD192, CD80 etc. – were estimated.
